# Genome sequence of the *Litoreibacter arenae* type strain (DSM 19593^T^), a member of the *Roseobacter* clade isolated from sea sand

**DOI:** 10.4056/sigs.4258318

**Published:** 2013-10-01

**Authors:** Thomas Riedel, Anne Fiebig, Jörn Petersen, Sabine Gronow, Nikos C. Kyrpides, Markus Göker, Hans-Peter Klenk

**Affiliations:** 1 UPMC Université Paris 6, UMR 7621, Observatoire Océanologique, Banyuls-sur-Mer, France; 2 CNRS, UMR 7621, LOMIC, Observatoire Océanologique, Banyuls-sur-Mer, France; 3 Leibniz Institute DSMZ – German Collection of Microorganisms and Cell Cultures Braunschweig, Germany; 4 DOE Joint Genome Institute, Walnut Creek, California, USA

**Keywords:** marine, rod-shaped, sea sand, sediment, motile, strictly aerobic, mesophile, chemoorganotrophic, halophilic, virus-like structures, carbon monoxide utilization, sulfur oxidation, *Rhodobacteraceae*, *Alphaproteobacteria*, *Thalassobacter arenae*

## Abstract

*Litoreibacter arenae* Kim *et al*. 2012 is a member of the genomically well-characterized *Rhodobacteraceae* clade within the *Roseobacter* clade. Representatives of this clade are known to be metabolically versatile and involved in marine carbon-producing and biogeochemical processes. They form a physiologically heterogeneous group of *Alphaproteobacteria* and were mostly found in coastal or polar waters, especially in symbiosis with algae, in microbial mats, in sediments or together with invertebrates and vertebrates. Here we describe the features of *L. arenae* DSM 19593^T^, including novel aspects of its phenotype, together with the draft genome sequence and annotation. The 3,690,113 bp long genome consists of 17 scaffolds with 3,601 protein-coding and 56 RNA genes. This genome was sequenced as part of the activities of the Transregional Collaborative Research Centre 51 funded by the German Research Foundation (DFG).

## Introduction

Strain GA2-M15^T^ (= DSM 19593 = KACC 12675) is the type strain of the species *Litoreibacter arenae* [1,2]. The genus *Litoreibacter* is a member of the highly abundant marine *Roseobacter* lineage, which plays an important role in the global carbon and sulfur cycles and thus for the climate on Earth. Phylogenetically, this alphaproteobacterial genus is related to the genera *Jannaschia, Octadecabacter* and *Thalassobius* [1]. Strain GA2-M15^T^ was isolated from a sea-sand sample from the coast of Homi Cape, Pohang City, South Korea as *Thalassobacter arenae* [1], which was later on reclassified into *Litoreibacter arenae* [2]. The name for the genus *Litoreibacter* was constructed from *litoreus* (‘belonging to the seashore’) and *bacter* (‘a rod’) [3]. The species epithet *arenae* refers to the Neolatin adjective *arenae*, ‘of sand’. Current PubMed records do not indicate any follow-up research with strain GA2-M15^T^ after the initial description of *T. arenae* [1] and its reclassification into *L. arenae* [2]. Here we present a summary classification and a set of features for *L. arenae* DSM 19593^T^, including novel aspects of its phenotype, together with the description of the genomic sequencing and annotation.

## Classification and features of the organism

### 16S rRNA gene analysis

A representative genomic 16S rRNA gene sequence of *L. arenae* DSM 19593^T^ was compared using NCBI BLAST [4,5] under default settings (e.g., considering only the high-scoring segment pairs (HSPs) from the best 250 hits) with the most recent release of the Greengenes database [6] and the relative frequencies of taxa and keywords (reduced to their stem [7]) were determined, weighted by BLAST scores. The most frequently occurring genera were *Jannaschia* (38.1%), *Thalassobacter* (15.4%), *Octadecabacter* (11.7%), *Roseovarius* (10.7%) and *Roseobacter* (10.2%) (28 hits in total). Regarding the three hits to sequences from other members of the genus, the average identity within HSPs was 96.0%, whereas the average coverage by HSPs was 98.7%. Among all other species, the one yielding the highest score was *'Octadecabacter orientus'* (DQ167247), which corresponded to an identity of 99.2% and an HSP coverage of 99.6%. (Note that the Greengenes database uses the INSDC (= EMBL/NCBI/DDBJ) annotation, which is not an authoritative source for nomenclature or classification). The highest-scoring environmental sequence was FJ664800 (Greengenes short name 'Quantitative dynamics cells plankton-fed microbial fuel cell clone plankton D11'), which showed an identity of 97.0% and an HSP coverage of 99.6%. The most frequently occurring keywords within the labels of all environmental samples which yielded hits were 'lake' (9.9%), 'tin' (9.8%), 'xiaochaidan' (9.4%), 'microbi' (2.6%) and 'sea' (2.5%) (222 hits in total). Environmental samples which yielded hits of a higher score than the highest scoring species were not found. 

**Figure 1:**
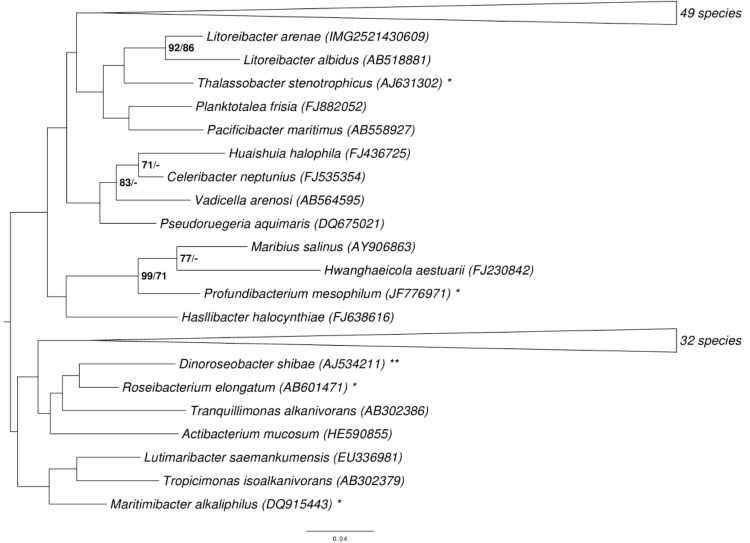
Phylogenetic tree highlighting the position of *L. arenae* relative to the type strains of the type species of the other genera within the family *Rhodobacteraceae*. The tree was inferred from 1,330 aligned characters [8,9] of the 16S rRNA gene sequence under the maximum likelihood (ML) criterion [10]. Rooting was done initially using the midpoint method [11] and then checked for its agreement with the current classification (Table 1). The branches are scaled in terms of the expected number of substitutions per site. Numbers adjacent to the branches are support values from 650 ML bootstrap replicates [12] (left) and from 1,000 maximum-parsimony bootstrap replicates [13] (right) if larger than 60%. Lineages with type strain genome sequencing projects registered in GOLD [14] are labeled with one asterisk, those also listed as 'Complete and Published' with two asterisks [15].

Figure 1 shows the phylogenetic neighborhood of *L. arenae* in a 16S rRNA sequence based tree. The sequence of the single 16S rRNA gene in the genome does not differ from the previously published 16S rDNA sequence (EU342372).

**Table 1:** Classification and general features of *L. arenae* DSM 19593^T^ according to the MIGS recommendations [16] published by the Genome Standards Consortium [17].

**MIGS ID**	**Property**	**Term**	**Evidence code**
	Current classification	Domain *Bacteria* Phylum *Proteobacteria* Class *Alphaproteobacteria* Order *Rhodobacterales* Family *Rhodobacteraceae* Genus *Litoreibacter*	TAS [18] TAS [19] TAS [20,21] TAS [21,22] TAS [21,23] TAS [2,3]
		Species *Litoreibacter arenae*	TAS [2]
MIGS-7	Subspecific genetic lineage (strain)	GA2-M15^T^	TAS [1]
MIGS-12	Reference for biomaterial	Kim *et al.* 2009	TAS [1]
	Gram stain	negative	TAS [1]
	Cell shape	rod-shaped	TAS [1]
			
	Motility	motile	TAS [1]
	Sporulation	non-sporulating	NAS
	Temperature range	mesophile (5°C-35°C)	TAS [1]
	Optimum temperature	30°C	TAS [1]
	Salinity	halophilic, 0.85-8% NaCl (w/v)	TAS [1]
MIGS-22	Relationship to oxygen	strictly aerobic	TAS [1]
	Carbon source	yeast extract, peptone	TAS [1]
MIGS-6	Habitat	sea sand, sediment, seawater	TAS [1]
MIGS-6.2	pH	6 – 9	TAS [1]
MIGS-15	Biotic relationship	free living	TAS [1]
	Biosafety level	1	TAS [24]
MIGS-23.1	Isolation	sea sand	TAS [1]
MIGS-4	Geographic location	Coast of Homi Cape, Pohang City, South Korea	TAS [1]
MIGS-4.1	Latitude	36.085	NAS
MIGS-4.2	Longitude	129.556	NAS
MIGS-4.3	Depth	not reported	

Evidence codes - TAS: Traceable Author Statement (i.e., a direct report exists in the literature); NAS: Non-traceable Author Statement (i.e., not directly observed for the living, isolated sample, but based on a generally accepted property for the species, or anecdotal evidence). Evidence codes are from the Gene Ontology project [25].

### Morphology and physiology

**Figure 2:**
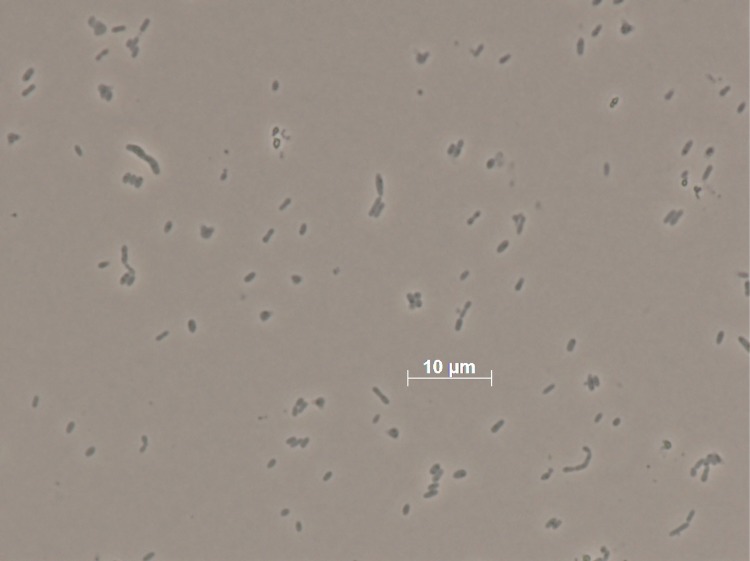
Micrograph of *L. arenae* DSM 19593^T^.

Cells of strain GA2-M15^T^ are Gram-negative short rods (0.7-1.2 µm in width and 1.2-2.4 µm in length) and contain a polar flagellum for motility [1], [Figure 2]. Polyhydroxybutyrate is accumulated in the cells. Colonies are deep-brown, circular and contain clear margins. Cells are catalase and oxidase positive [1]. Growth occurs at 5-35 °C with an optimum at 30 °C. Cells were successfully grown on marine agar (MA), nutrient agar (NA, weak growth), salt tolerance agar (STA, containing 1% (w/v) tryptone, 0.3% (w/v) yeast extract and 1.5% (w/v) agar supplemented with salts) as well as on basal medium agar (BMA, recipe after [1]). No growth was observed on Reasoner’s 2A agar (R2A), trypticase soy agar (TSA) or MacConkey agar. The salinity range for growth is 0.85–8% NaCl (w/v), but the strain does not grow below 0.34% or at above 10% NaCl (w/v). The pH range for growth is pH 6–9 with an optimum at pH 7 [1].

Cells hydrolyze aesculin and tyrosine weakly, but do not show any hydrolysis of alginic acid, casein, chitin, CM-cellulose, DNA, gelatin, pectin, starch or urea [1]. They assimilate citrate, D-fructose, D-galactose, D-glucose, L-glutamate, glycerol, *β*-hydroxybutyrate, D-mannitol, D-mannose, melibiose, propionate, pyruvate, L-serine, L-tyrosine and D-xylose, but not L-alanine, L-arabinose, L-aspartate, cellobiose, glycine, L-histidine, lactose, L-leucine, maltose, L-rhamnose, D-ribose, sucrose, L-threonine or trehalose. Cells are positive for *β*-galactosidase, esterase (C4), esterase lipase (C8), leucine arylamidase, naphthol-AS-BI-phosphohydrolase, *α*-glucosidase and *β*-glucosidase, but negative for indole production, arginine dihydrolase, alkaline phosphatase, lipase (C14), valine arylamidase, cystine arylamidase, trypsin, *α*-chymotrypsin, acid phosphatase, *α*-galactosidase, *β*-glucuronidase, *N*-acetyl-*β*-glucosaminidase, *α*-mannosidase and *α*-fucosidase [1].

The substrate utilization and resistance patterns of *L. arenae* DSM 19593^T^ were also determined for this study using Generation-III microplates in an OmniLog phenotyping device (BIOLOG Inc., Hayward, CA, USA). The microplates were inoculated at 28°C with a cell suspension at a cell density of 95-96% turbidity and dye IF-A. Further additives were vitamin, micronutrient and sea salt solutions. The exported measurement data were further analyzed with the opm package for R [26,27], using its functionality for statistically estimating parameters from the respiration curves such as the maximum height, and automatically translating these values into negative, ambiguous, and positive reactions. The strain was studied in two independent biological replicates, and reactions with a different behavior between the two repetitions, if any, were regarded as ambiguous.

*L. arenae* DSM 19593^T^ showed positive reactions for pH 6, 1% NaCl, 4% NaCl, D-galactose, 3-O-methyl-D-glucose, D-fucose, L-fucose, L-rhamnose, 1% sodium lactate, *myo*-inositol, rifamycin SV, L-aspartic acid, L-glutamic acid, L-histidine, L-serine, D-glucuronic acid, glucuronamide, quinic acid, L-lactic acid, citric acid, *α*-keto-glutaric acid, D-malic acid, L-malic acid, nalidixic acid, lithium chloride, acetic acid and sodium formate.

No reaction was found for dextrin, D-maltose, D-trehalose, D-cellobiose, *β*-gentiobiose, sucrose, D-turanose, stachyose, pH 5, D-raffinose, *α*-D-lactose, D-melibiose, *β*-methyl-D-galactoside, D-salicin, *N*-acetyl-D-glucosamine, *N*-acetyl-*β*-D-mannosamine, *N*-acetyl-D-galactosamine, *N*-acetyl-neuraminic acid, 8% NaCl, D-glucose, D-mannose, D-fructose, inosine, fusidic acid, D-serine, D-sorbitol, D-mannitol, D-arabitol, glycerol, D-glucose-6-phosphate, D-fructose-6-phosphate, D-aspartic acid, D-serine, troleandomycin, minocycline, gelatin, glycyl-L-proline, L-alanine, L-arginine, L-pyroglutamic acid, lincomycin, guanidine hydrochloride, niaproof, pectin, D-galacturonic acid, L-galactonic acid-*γ*-lactone, D-gluconic acid, mucic acid, D-saccharic acid, vancomycin, tetrazolium violet, tetrazolium blue, *p*-hydroxy-phenylacetic acid, methyl pyruvate, D-lactic acid methyl ester, bromo-succinic acid, potassium tellurite, tween 40, *γ*-amino-n-butyric acid, *α*-hydroxy-butyric acid, *β*-hydroxy-butyric acid, *α*-keto-butyric acid, acetoacetic acid, propionic acid, aztreonam, butyric acid and sodium bromate.

The measured utilization of carbon sources differs in some aspects from the one recorded in [1]. L-histidine and L-rhamnose were reported in [1] not to support bacterial growth, whereas in the Omnilog measurements both substrates yielded a positive reaction. This may be due to the higher sensitivity of respiratory measurements [28]. The utilization of propionate, D-fructose, D-glucose, D-mannose, D-mannitol, melibiose and glycerol reported by [1] could not be confirmed by the Omnilog measurements. Changes in the substrate-utilization pattern may arise from distinct cultivation conditions such as growth medium and temperature.

### Chemotaxonomy

The principal cellular fatty acids of strain GA2-M15^T^ are C_18:1 ω7c_ (74.3%), C_16:0_ (10.4%), C_18:1 ω7c 11-methyl_ (5.9%), C_10:0 3-OH_ (3.7%) as well as an unknown fatty acid 11.799 (3.0%) [1]. In comparison to *Thalassobacter stenotrophicus* DSM 16310^T^ [29,30], strain GA2-M15^T^ reflected a higher content of C_16:0_ (1.1% vs 10.4%) [1]. The predominant polar lipids are diphosphatidylglycerol, phosphatidylglycerol, phosphatidylethanolamine and phosphatidyl-choline [1].

## Genome sequencing and annotation

### Genome project history

The strain was first chosen for genome sequencing in the *Genomic Encyclopedia of Bacteria and Archaea* (GEBA) phase I project according the GEBA criteria [31,32], but then sequenced as part of the “Ecology, Physiology and Molecular Biology of the *Roseobacter* clade project: Towards a Systems Biology Understanding of a Globally Important Clade of Marine Bacteria” funded by the German Research Council (DFG). Project information is stored in the Genomes OnLine Database [14]. The Whole Genome Shotgun (WGS) sequence is deposited in Genbank and the Integrated Microbial Genomes database (IMG) [33]. A summary of the project information is shown in Table 2.

**Table 2:** Genome sequencing project information

**MIGS ID**	**Property**	**Term**
MIGS-31	Finishing quality	Non-contiguous finished
MIGS-28	Libraries used	One Illumina PE library (520 bp insert size)
MIGS-29	Sequencing platforms	Illumina GA II×, Illumina MiSeq
MIGS-31.2	Sequencing coverage	195 × Illumina
MIGS-30	Assemblers	velvet version 1.1.36, consed version 20.0
MIGS-32	Gene calling method	Prodigal
	GOLD ID	Gi11991
	NCBI project ID	178144
	INSDC ID	AONI00000000
	Genbank Date of Release	March 13, 2013
	IMG Taxon OID	2518285519
MIGS-13	Source material identifier	DSM 19593
	Project relevance	Tree of Life, biodiversity

### Growth conditions and DNA isolation

A culture of DSM 19593^T^ was grown aerobically in DSMZ medium 514 [34] at 28°C. Genomic DNA was isolated using Jetflex Genomic DNA Purification Kit (GENOMED 600100) following the standard protocol provided by the manufacturer but modified by an incubation time of 60 min, incubation on ice over night on a shaker, the use of additional 50 µl proteinase K, and the addition of 100 µl protein precipitation buffer. DNA is available from DSMZ through the DNA Bank Network [35].

### Genome sequencing and assembly

The genome was sequenced using one Illumina PE library (Table 2). Illumina sequencing [36] was performed on a GA IIx platform with 150 cycles. The paired-end library contained 520 bp insert size. To correct sequencing errors and improve quality of the reads, clipping was performed using fastq-mcf [37] and quake [38]. After this step 4,717,610 reads with a median length of 124 bp were assembled using velvet [39]. The resulting draft genome consisted of 71 contigs organized in 45 scaffolds. The initial draft sequences were separated into artificial Sanger reads of 1,000 nt size plus 75 nt overlap. The number of gaps was reduced by manual editing in phred/phrap/consed version 20.0 [40]. The final assembly was composed of 17 contigs organized in 15 scaffolds. (The version deposited at Genbank contains two scaffolds less, which did not meet the requirements for the minimal contig length.) The additional fragments 'thalar_Contig12.1' and 'thalar_Contig18_1.4' can be found in the IMG database).The combined sequences provided a 195× coverage of the genome.

### Genome annotation

Genes were identified using Prodigal [41] as part of the JGI genome annotation pipeline [42]. The predicted CDSs were translated and used to search the National Center for Biotechnology Information (NCBI) nonredundant database, UniProt, TIGR-Fam, Pfam, PRIAM, KEGG, COG, and InterPro databases. Identification of RNA genes were carried out by using HMMER 3.0rc1 [43] (rRNAs) and tRNAscan-SE 1.23 [44] (tRNAs). Other non-coding genes were predicted using INFERNAL 1.0.2 [45]. Additional gene prediction analysis and functional annotation was performed within the Integrated Microbial Genomes - Expert Review (IMG-ER) platform [33]. CRISPR elements were detected using CRT [46] and PILER-CR [47].

## Genome properties

**Figure 3:**
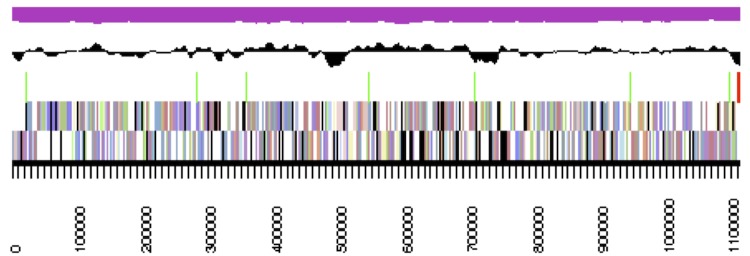
Graphical map of the largest scaffold. From bottom to top: genes on forward strand (color by COG categories), genes on reverse strand (color by COG categories), RNA genes (tRNAs green, rRNAs red, other RNAs black), GC content, GC skew.

The genome statistics are provided in Table 3 and Figure 3. The genome consists of a 3.56 Mbp chromosome and a G+C content of 60% and a 140 kbp plasmid with a G+C content of 59%. Of the 3,657 genes predicted, 3,601 were protein-coding genes, and 56 RNAs. The majority of the protein-coding genes (81.8%) were assigned a putative function while the remaining ones were annotated as hypothetical proteins. The distribution of genes into COGs functional categories is presented in Table 4.

**Table 3:** Genome Statistics

**Attribute**	**Number**	**% of Total**
Genome size (bp)	3,690,113	100.00
DNA coding region (bp)	3,376,611	91.50
DNA G+C content (bp)	2,222,524	60.23
Number of scaffolds	17	
Extrachromosomal elements	1	
Total genes	3,657	100.00
RNA genes	56	1.53
rRNA operons	1	
tRNA genes	43	1.18
Protein-coding genes	3,601	98.47
Genes with function prediction (proteins)	2,990	81.76
Genes in paralog clusters	1,040	28.44
Genes assigned to COGs	2,873	78.56
Genes assigned Pfam domains	3,047	83.32
Genes with signal peptides	347	9.49
Genes with transmembrane helices	836	22.86
CRISPR repeats	0	

**Table 4:** Number of genes associated with the general COG functional categories

**Code**	**Value**	**%age**	**Description**
J	164	5.2	Translation, ribosomal structure and biogenesis
A	0	0.0	RNA processing and modification
K	197	6.3	Transcription
L	135	4.3	Replication, recombination and repair
B	3	0.1	Chromatin structure and dynamics
D	32	1.0	Cell cycle control, cell division, chromosome partitioning
Y	0	0.0	Nuclear structure
V	46	1.5	Defense mechanisms
T	97	3.1	Signal transduction mechanisms
M	176	5.6	Cell wall/membrane/envelope biogenesis
N	32	1.0	Cell motility
Z	2	0.1	Cytoskeleton
W	0	0.0	Extracellular structures
U	73	2.3	Intracellular trafficking and secretion, and vesicular transport
O	121	3.8	Posttranslational modification, protein turnover, chaperones
C	195	6.2	Energy production and conversion
G	200	6.4	Carbohydrate transport and metabolism
E	358	11.4	Amino acid transport and metabolism
F	82	2.6	Nucleotide transport and metabolism
H	138	4.4	Coenzyme transport and metabolism
I	146	4.6	Lipid transport and metabolism
P	141	4.5	Inorganic ion transport and metabolism
Q	99	3.1	Secondary metabolites biosynthesis, transport and catabolism
R	410	13.0	General function prediction only
S	303	9.6	Function unknown
-	784	21.4	Not in COGs

## Insights into the genome

The genome comprises a single extrachromosomal element (with not yet validated circularity), 'thalar_Contig204.17', 139.9 kbp in size containing 130 protein-coding genes including a large RTX-toxin gene and a F0F1-type ATPase operon. It contains the typical replication modules. Its replication system is of the ABC-9 type with the compatibility group RepC-9 [48]. This type of repABC operon was found in two representatives of the genera *Octadecabacter* and *Roseobacter*, respectively, as well as in *Dinoroseobacter shibae* [48]. The presence of the replication-initiation gene DnaA (Thalar_03034) reveals the chromosomal origin of largest, 1.091 Mbp long scaffold 'thalar_Contig148.14', but it harbors neither a plasmid stability module nor a type IV secretion system.

Genome analysis of strain DSM 19593^T^ revealed the presence of genes encoding proteins associated to carbon monoxide utilization (thalar_00241, thalar_00242, thalar_02265, thalar_03324, thalar_03325, thalar_03395, thalar_03397) as well as genes forming a putative operon, which are involved in the oxidation of sulfur (thalar_01786 to_01792) indicating the oxidation of sulfur to produce energy. Additional gene sequences of interest encode a homogentisate 1,2-dioxygenase (thalar_03573), several haloacid dehalogenase superfamily proteins (thalar_00489, thalar_00580, thalar_01120, thalar_01943, thalar_02401) and a 2-haloalkanoic acid dehalogenase type II (thalar_00287). The presence of such genes could indicate a respiratory degradation of recalcitrant compounds by strain DSM 19593^T^ in its ecological niche.

Further genes encoding a N-acyl-L-homoserine lactone synthetase (thalar_00160) and a response regulator (thalar_00161) associated to quorum sensing were observed [49-52]. Genome analysis of strain DSM 19593^T^ also revealed the presence of genes encoding a bacteriophage associated genes (e.g., thalar_00003 to 00007). A gene encoding a sensor of blue light using FAD (BLUF, thalar_02670) was also detected, indicating possible blue-light dependent signal transduction.

## References

[1] Kim BY Weon HY Son JY Lee CM Hong SB Jeon YA Koo BS Kwon SW *Thalassobacter arenae* sp. nov., isolated from sea sand in Korea. Int J Syst Evol Microbiol 2009; 59:487-490 10.1099/ijs.0.65841-0 19244427

[2] Kim YO Park S Nam BH Kang SJ Hur YB Kim DG Oh TK Yoon JH Description of *Litoreibacter meonggei* sp. nov., isolated from the sea squirt *Halocynthia roretzi*, reclassification of *Thalassobacter arenae* as *Litoreibacter arenae* comb. nov. and emended description of the genus *Litoreibacter* Romanenko et al. 2011. Int J Syst Evol Microbiol 2012; 62:1825-1831 10.1099/ijs.0.035113-0 21984668

[3] Romanenko LA Tanaka N Frolova GM Svetashev VI Mikailov VV *Litoreibacter albidus* gen. nov., sp. nov. and *Litoreibacter janthinus* sp. nov., members of the *Alphaproteobacteria* isolated from the seashore. Int J Syst Evol Microbiol 2011; 61:148-154 10.1099/ijs.0.019513-0 20173002

[4] Altschul SF Gish W Miller W Myers EW Lipman DJ Basic local alignment search tool. J Mol Biol 1990; 215:403-410 2231712 10.1016/S0022-2836(05)80360-2

[5] Korf I, Yandell M, Bedell J. BLAST, O'Reilly, Sebastopol, 2003.

[6] DeSantis TZ Hugenholtz P Larsen N Rojas M Brodie EL Keller K Huber T Dalevi D Hu P Andersen GL Greengenes, a Chimera-Checked 16S rRNA Gene Database and Workbench Compatible with ARB. Appl Environ Microbiol 2006; 72:5069-5072 10.1128/AEM.03006-05 16820507 PMC1489311

[7] Porter MF. An algorithm for suffix stripping. *Program: electronic library and information systems* 1980; **14**:130-137.

[8] Lee C Grasso C Sharlow MF Multiple sequence alignment using partial order graphs. Bioinformatics 2002; 18:452-464 10.1093/bioinformatics/18.3.452 11934745

[9] Castresana J Selection of conserved blocks from multiple alignments for their use in phylogenetic analysis. Mol Biol Evol 2000; 17:540-552 10.1093/oxfordjournals.molbev.a026334 10742046

[10] Stamatakis A Hoover P Rougemont J A rapid bootstrap algorithm for the RAxML web-servers. Syst Biol 2008; 57:758-771 10.1080/10635150802429642 18853362

[11] Hess PN De Moraes Russo CA An empirical test of the midpoint rooting method. Biol J Linn Soc Lond 2007; 92:669-674 10.1111/j.1095-8312.2007.00864.x PMC7110036 32287391

[12] Pattengale ND Alipour M Bininda-Emonds ORP Moret BME Stamatakis A How Many Bootstrap Replicates Are Necessary? Lect Notes Comput Sci 2009; 5541:184-200 10.1007/978-3-642-02008-7_13

[13] Swofford DL. PAUP*: Phylogenetic Analysis Using Parsimony (*and Other Methods), Version 4.0 b10. Sinauer Associates, Sunderland, 2002.

[14] Pagani I Liolios K Jansson J Chen IM Smirnova T Nosrat B Markowitz VM Kyrpides NC The GenomesOnLine Database (GOLD) v.4: status of genomic and metagenomic projects and their associated metadata. Nucleic Acids Res 2012; 40:D571-D579 10.1093/nar/gkr1100 22135293 PMC3245063

[15] Wagner-Döbler I Ballhausen B Berger M Brinkhoff T Buchholz I Bunk B Cypionka H Daniel R Drepper T Gerdts G The complete genome sequence of the algal symbiont *Dinoroseobacter shibae* – a hitchhiker's guide to life in the sea. ISME J 2010; 4:61-77 10.1038/ismej.2009.94 19741735

[16] Field D Garrity G Gray T Morrison N Selengut J Sterk P Tatusova T Thomson N Allen MJ Angiuoli SV The minimum information about a genome sequence (MIGS) specification. Nat Biotechnol 2008; 26:541-547 10.1038/nbt1360 18464787 PMC2409278

[17] Field D Amaral-Zettler L Cochrane G Cole JR Dawyndt P Garrity GM Gilbert J Glöckner FO Hirschman L Karsch-Mzrachi I PLoS Biol 2011; 9:e1001088 10.1371/journal.pbio.1001088 21713030 PMC3119656

[18] Woese CR Kandler O Weelis ML Towards a natural system of organisms. Proposal for the domains *Archaea* and *Bacteria.* Proc Natl Acad Sci USA 1990; 87:4576-4579 10.1073/pnas.87.12.4576 2112744 PMC54159

[19] Garrity GM, Bell JA, Lilburn T. Phylum XIV. *Proteobacteria* phyl nov. In: Brenner DJ, Krieg NR, Stanley JT, Garrity GM (eds), Bergey’s Manual of Sytematic Bacteriology, second edition. Vol. 2 (The *Proteobacteria*), part B (The *Gammaproteobacteria*), Springer, New York, 2005, p. 1.

[20] Garrity GM, Bell JA, Lilburn T. Class I. *Alphaproteobacteria* class. nov. In: Brenner DJ, Krieg NR, Stanley JT, Garrity GM (eds), Bergey’s Manual of Sytematic Bacteriology, second edition. Vol. 2 (The *Proteobacteria*), part C (The *Alpha*-, *Beta*-, *Delta*-, and *Epsilonproteobacteria*), Springer, New York, 2005, p. 1.

[21] Validation List No 107. List of new names and new combinations previously effectively, but not validly, published. Int J Syst Evol Microbiol 2006; 56:1-6 10.1099/ijs.0.64188-0 16403855

[22] Garrity GM, Bell JA, Lilburn T. Order III. *Rhodobacterales* ord. nov. *In*: Brenner DJ, Krieg NR, Staley JT, Garrity GM (eds), Bergey’s Manual of Systematic Bacteriology, second edition. vol. 2 (The *Proteobacteria*), part C (The *Alpha-, Beta-, Delta-*, and *Epsilonproteobacteria*), Springer, New York, 2005, p. 161.

[23] Garrity GM, Bell JA, Lilburn T. Family I. *Rhodobacteraceae* fam. nov. *In*: Brenner DJ, Krieg NR, Staley JT, Garrity GM (eds), Bergey’s Manual of Systematic Bacteriology, second edition. vol. 2 (The *Proteobacteria*), part C (The *Alpha-, Beta-, Delta-*, and *Epsilonproteobacteria*), Springer, New York, 2005, p. 161.

[24] BAuA Classification of *Bacteria* and *Archaea* in risk groups. TRBA 2010; 466:93

[25] Ashburner M Ball CA Blake JA Botstein D Butler H Cherry JM Davis AP Dolinski K Dwight SS Eppig JT Gene ontology: tool for the unification of biology. The Gene Ontology Consortium. Nat Genet 2000; 25:25-29 10.1038/75556 10802651 PMC3037419

[26] Vaas LA Sikorski J Michael V Göker M Klenk HP Visualization and curve-parameter estimation strategies for efficient exploration of phenotype microarray kinetics. PLoS ONE 2012; 7:e34846 10.1371/journal.pone.0034846 22536335 PMC3334903

[27] Vaas LA Sikorski J Hofner B Fiebig A Buddruhs N Klenk HP Göker M opm: an R package for analyzing OmniLog® phaenotype microarray date. Bioinformatics 2013; 29:1823-1824 10.1093/bioinformatics/btt291 23740744

[28] Bochner BR Global phenotypic characterization of bacteria. FEMS Microbiol Rev 2009; 33:191-205 10.1111/j.1574-6976.2008.00149.x 19054113 PMC2704929

[29] Macián MC Arahal DR Garay E Ludwig W Schleifer KH Pujalte MJ *Thalassobacter stenotrophicus* gen. nov., sp. nov., a novel marine alphaproteobacterium isolated from Mediterranean sea water. Int J Syst Evol Microbiol 2005; 55:105-110 10.1099/ijs.0.63275-0 15653862

[30] Pujalte MJ Macián MC Arahal DR Garay E *Thalassobacter stenothrophicus* Macian et al 2005 is a later synonym of *Jannaschia cystaugens* Adachi et al. 2004, with emended description of the genus *Thalassobacter**.* Int J Syst Evol Microbiol 2005; 55:1959-1963 10.1099/ijs.0.63617-0 16166695

[31] Göker M Klenk HP Phylogeny-driven target selection for genome-sequencing (and other) projects. Stand Genomic Sci 2013; 8:360-374 10.4056/sigs.3446951 23991265 PMC3746418

[32] Wu D Hugenholtz P Mavromatis K Pukall R Dalin E Ivanova NN Kunin V Goodwin L Wu M Tindall BJ A phylogeny-driven Genomic Encyclopaedia of *Bacteria* and *Archaea.* Nature 2009; 462:1056-1060 10.1038/nature08656 20033048 PMC3073058

[33] Markowitz VM Ivanova NN Chen IMA Chu K Kyrpides NC IMG ER: a system for microbial genome annotation expert review and curation. Bioinformatics 2009; 25:2271-2278 10.1093/bioinformatics/btp393 19561336

[34] List of growth media used at the DSMZ: http://www.dmsz.de/catalogues/cataloque-microorganisms/culture-technology/list-of-media-for-microorganisms.html

[35] Gemeinholzer B Dröge G Zetzsche H Haszprunar G Klenk HP Güntsch A Berendsohn WG Wägele JW The DNA Bank Network: the start from a German initiative. Biopreserv Biobank 2011; 9:51-55 10.1089/bio.2010.0029 24850206

[36] Bennett S Solexa Ltd. Pharmacogenomics 2004; 5:433-438 10.1517/14622416.5.4.433 15165179

[37] Aronesty E. *ea-utils*: Command-line tools for processing biological sequencing data; 2011 http://code.google.com/p/ea-utils

[38] Kelley DR Schatz MC Salzberg SL Quake: quality-aware detection and correction of sequencing errors. Genome Biol 2010; 11:R116 10.1186/gb-2010-11-11-r116 21114842 PMC3156955

[39] Zerbino DR Birney E Velvet: algorithms for de novo short read assembly using de Bruijn graphs. Genome Res 2008; 18:821-829 10.1101/gr.074492.107 18349386 PMC2336801

[40] Gordon D Abajian C Green P Consed: a graphical tool for sequence finishing. Genome Res 1998; 8:195-202 10.1101/gr.8.3.195 9521923

[41] Hyatt D Chen GL LoCascio PF Land ML Larimer FW Hauser LJ Prodigal: prokaryotic gene recognition and translation initiation site identification. BMC Bioinformatics 2010; 11:119 10.1186/1471-2105-11-119 20211023 PMC2848648

[42] Mavromatis K Ivanova NN Chen IM Szeto E Markowitz VM Kyrpides NC The DOE-JGI Standard operating procedure for the annotations of microbial genomes. Stand Genomic Sci 2009; 1:63-67 10.4056/sigs.632 21304638 PMC3035208

[43] Finn DR, Clements J, Eddy SR. HMMER web server: interactive sequence similarity searching. Nucleic Acids Research 2011, Web Server Issue **39**:W29-W37. 10.1093/nar/gkr367 PMC3125773 21593126

[44] Lowe TM Eddy SR tRNAscan-SE: A Program for Improved Detection of Transfer RNA Genes in Genomic Sequence. Nucleic Acids Res 1997; 25:955-964 9023104 10.1093/nar/25.5.955 PMC146525

[45] Nawrocki EP Kolbe DL Eddy SR Infernal 1.0: Inference of RNA alignments. Bioinformatics 2009; 25:1335-1337 10.1093/bioinformatics/btp157 19307242 PMC2732312

[46] Bland C Ramsey TL Sabree F Lowe M Brown K Kyrpides NC Hugenholtz P CRISPR recognition tool (CRT): a tool for automatic detection of clustered regularly interspaced palindromic repeats. BMC Bioinformatics 2007; 8:209 10.1186/1471-2105-8-209 17577412 PMC1924867

[47] Edgar RC PILER-CR: Fast and accurate identification of CRISPR repeats. BMC Bioinformatics 2007; 8:18 10.1186/1471-2105-8-18 17239253 PMC1790904

[48] Petersen J Brinkmann H Pradella S Diversity and evolution of repABC type plasmids in *Rhodobacterales.* Environ Microbiol 2009; 11:2627-2638 10.1111/j.1462-2920.2009.01987.x 19601964

[49] Wagner-Döbler I Thiel V Eberl L Allgaier M Bodor A Meyer S Ebner S Hennig A Pukall R Schulz S Discovery of complex mixtures of novel long-chain quorum sensing signals in free-living and host-associated marine alphaproteobacteria. ChemBioChem 2005; 6:2195-2206 10.1002/cbic.200500189 16283687

[50] Bassler BL How bacteria talk to each other: regulation of gene expression by quorum sensing. Curr Opin Microbiol 1999; 2:582-587 10.1016/S1369-5274(99)00025-9 10607620

[51] Henke JM Bassler BL Bacterial social engagements. Trends Cell Biol 2004; 14:648-656 10.1016/j.tcb.2004.09.012 15519854

[52] Waters CM Bassler BL Quorum Sensing: Cell-to-Cell Communication in Bacteria. Annu Rev Cell Dev Biol 2005; 21:319-346 10.1146/annurev.cellbio.21.012704.131001 16212498

